# Design of mobile and website health application devices for drug tolerability in hereditary fructose intolerance

**DOI:** 10.1186/s13023-023-03011-x

**Published:** 2024-01-05

**Authors:** Elsa Izquierdo-García, Andrea Lázaro-Cebas, Berta Montero Pastor, Ana Such Díaz, Elena Alba Álvaro-Alonso, Laura López Guerra, Ismael Escobar-Rodríguez

**Affiliations:** 1 Pharmacy Department, Hospital Universitario Infanta Leonor, Av. Gran Vía del Este, 80, Madrid, 28031 Spain; 2 Pharmacy Department, Hospital General Universitario Santa Lucía, Cartagena, Spain

**Keywords:** Hereditary Fructose Intolerance, Rare Diseases, Excipients, Medical informatics applications, e-Health, m-Health

## Abstract

**Background:**

Hereditary fructose intolerance (HFI) is a rare metabolic disease caused by aldolase B deficiency. The aim of our study was to analyse excipient tolerability in patients with HFI and other related diseases and to design mobile and website health applications to facilitate the search for drugs according to their tolerance.

**Results:**

A total of 555 excipients listed in the Spanish Medicines Agency database (July 2023) were classified as suitable for HFI patients, suitable with considerations ((glucose and glucose syrup, intravenous sucrose, oral mannitol, polydextrose, gums and carrageenans, ethanol, sulfite caramel and vanilla), not recommended (intravenous mannitol) and contraindicated (fructose, oral sucrose, invert sugar, sorbitol, maltitol, lactitol, isomaltitol, fruit syrups, honey, sucrose esters and sorbitol esters). Glucose and glucose syrup were classified as suitable with considerations due to its possible fructose content and their potential endogenous fructose production. For other related intolerances, wheat starch was contraindicated and oatmeal was not recommended in celiac disease; oral lactose and lactose-based coprocessed excipient (Cellactose®) were not recommended in lactose intolerance; and glucose, invert sugar and oral sucrose were not recommended in diabetes mellitus. The applications were named IntoMed®. Results are listed in order of tolerability (suitable drugs appear first and contraindicated drugs at the end), and they are accompanied by a note detailing their classified excipients. If a drug contains excipients within different categories, the overall classification will be the most restrictive. The apps are also able to classify substances with the same criteria if they act as active ingredients. The tools exhibited good usability (82.07 ± 13.46 points on the System Usability Scale [range: 0-100]) on a sample of HFI patients, their families and health care professionals.

**Conclusions:**

IntoMed® is a tool for finding information about the tolerability of drugs according to excipients for patients with HFI and other related intolerances, with good usability. It is a fast and reliable system that covers the current excipient legislation and expands on it with other specific information: HFI patients should be alert for excipients such as mannitol (especially in intravenous drugs), fruit syrups, honey, sulfite caramel or vanilla. Glucose might contain or produce fructose, and special precaution is needed because of potential errors in their composition.

## Introduction

Hereditary fructose intolerance (HFI) (OMIM #229,600) is a rare autosomal recessive metabolic disorder with a prevalence of 1–9 per 100,000 in Europe. It is caused by an enzymatic deficiency of aldolase B, an enzyme responsible for fructose metabolism (mainly in the liver). Following a large ingestion of fructose, these patients develop nausea, vomiting, hepatic and renal dysfunction, postprandial hypoglycaemia or even death. Moreover, chronic consumption of small amounts of fructose results in long-term effects (feeding difficulties, occasional vomiting, hepatic disorders and growth retardation) and, in some cases, even persistent symptoms, such as nonalcoholic fatty liver disease (NAFLD), hepatomegaly [1, 2] and altered kidney and vascular function, even with a fructose-restricted diet [3]. Currently, the only available treatment is the reduction of all fructose sources in the diet to less than 20–40 mg of fructose/kg/day (maximum of 1–2 g fructose/day); however, there is a lack of expert consensus [4].

In addition to foods that naturally contain fructose (fruits, some vegetables, honey, etc.), other sugars and sweeteners should be avoided, such as sucrose (fructose and glucose disaccharide), sorbitol (sorbitol dehydrogenase converts sorbitol to fructose), sweeteners containing sorbitol (such as maltitol, lactitol or isomaltitol), and tagatose (a fructose isomer that is metabolised by aldolase B) [4, 5].

In medicines, sugars and sweeteners are widely used in oral formulations (syrups, suspensions, tablets, etc.). This kind of excipient, especially sorbitol, can also be found in parenteral formulations and is mainly used as a stabiliser in protein/peptide drugs such as antibodies or vaccines. According to the current European medicines legislation [6], all excipients should appear on the labelling and package leaflet, and a warning for HFI patients is mandatory in drugs containing fructose, sorbitol, sucrose, invert sugar, isomaltitol, lactitol and maltitol. In addition, intravenous (IV) drugs with fructose and sorbitol are contraindicated in all children under two years because they may not yet be diagnosed with HFI (Table 1). On the other hand, there are other excipients, such as stabilisers, preservatives, flavours, colours, etc., that, due to their composition or metabolism, could generate uncertainty for patients, caregivers or health care professionals regarding their possible administration in HFI patients.

In this situation, it can be difficult to obtain fast, accessible and consensus information about the tolerance of different excipients in patients with HFI. In fact, the ease of finding this information is one of the demands of HFI patients regarding medication use [7]. In addition, HFI patients may have other intolerances, making it difficult to select appropriate medicines. In some studies, type 2 diabetes mellitus [8, 9] and celiac disease have been associated with several genetic and immune disorders, including HFI [10]. Other food intolerances, such as lactose intolerance, are frequent in the general population and may be present in these patients.

The potential of mobile device applications (apps) for rare diseases [11] or for food-related conditions is increasing. There are already apps that help patients identify food allergens or substances causing food intolerance, [12, 13] but there are no apps specialising in drug tolerability.

The aim of the present study was to analyse excipient tolerability in HFI patients and to design a mobile and website health application to allow patients with HFI and other related intolerances to identify and classify drugs according to tolerability.

**Table 1:** Information about drug excipients contraindicated in HFI patients from labelling and package leaflets (EMA/CHMP/302,620/2017).

Excipient	Route of administration	Threshold	Comments on the labelling
Fructose Sorbitol (E 420)	Intravenous (IV)	Zero	Patients with hereditary fructose intolerance (HFI) must not be given this medicine unless strictly necessary. Babies and young children (below two years of age) may not yet be diagnosed with HFI. Medicines containing fructose/sorbitol given intravenously may be life-threatening and are contraindicated in this population unless there is an overwhelming clinical need and no alternatives are available. A detailed history of HFI symptoms has to be taken for each patient prior to being given this medicinal product.
	Oral, parenteral (other than IV)	5 mg/kg/day	Patients with rare hereditary problems of fructose intolerance should not take this medicine.
Invert sugar	Oral	Zero	
Isomaltitol (isomalt) (E 953)			
Lactitol (E 966)			
Maltitol (E 965), maltitol liquid (hydrogenated glucose syrup)			
Sucrose			

## Methods

The study was developed in two phases. First, excipient tolerability in patients with HFI and other intolerances was analysed, and second, the mobile device application and website functionalities were designed. Then, their usability was evaluated.

### Excipient classification

All excipients listed in the Spanish Medicines Agency database [14] in July 2023 were analysed. The database includes all authorised and marketed medicines in Spain and their technical information, including excipients with a known action or effect.

All excipients were classified into four categories according to their tolerance and the administration route (parenteral, oral and topical).

In HFI patients, the four groups were as follows:

- Suitable: excipients that are eliminated unaltered, metabolised by a route other than aldolase B and/or those without fructose, sucrose or sorbitol in their composition.
- Suitable with some considerations: excipients suitable for HFI patients, although some caution should be exercised.
- Not recommended: excipients with an unknown metabolism or composition or lack of expert consensus about their tolerance in HFI patients.
- Contraindicated: excipients that contain fructose, sucrose or sorbitol or are metabolised into these compounds.

The current European legislation was used to classify the list of excipients for celiac disease, lactose intolerance and diabetes mellitus [6] using the same classifications as those for HFI.

### Application design and usability

Both the website and mobile device app were designed by a multidisciplinary team composed of hospital pharmacists with experience in HFI management, software engineers, HFI patients and HFI family members, who defined the contents and utilities. All the medicines marketed as of July 23 that appeared in the Spanish Medicines Agency database were classified into four categories according to the tolerability of the excipients with a colour and face scale: suitable (green), suitable with considerations (blue), not recommended (orange) and contraindicated (red). If a drug contained excipients with different categories, the overall classification was the most restrictive. If a new excipient appears in the Spanish Medicine Agency database, the drug will be unclassified until a pharmacist reviews the tolerability of its excipients.

After the functional requirements were designed, the apps were developed by the informatics company. Before the apps were launched, usability was evaluated with a validated Spanish version of the System Usability Scale (SUS) for the assessment of electronic tools [15]. The evaluation was carried out in a sample of HFI patients or their family members through the Spanish HFI association and health care professionals not directly related to the tool design. The SUS is a Likert-type 10-item questionnaire with positive and negative questions each scored from 1 to 5 (from strongly disagree to strongly agree, respectively). The score contribution for the positive questions is the scale position minus 1, and the contribution for the negative questions is 5 minus the scale position; the overall score was calculated from the sum of all item scores multiplied by 2.5 (range: 0 to 100). Tools with a score between 68 and 84 points are considered to have good usability, and those with a score above 85 have excellent usability [15, 16].

## Results

### Excipient classification

A total of 555 excipients were listed in the Spanish Medicines Agency database as of July 2023. First, the excipients were classified into four categories: suitable, suitable with some considerations (Table 2), not recommended (Table 3) and contraindicated (Table 4) in HFI patients.

**Table 2:** Suitable excipients with some considerations for HFI patients

Suitable excipients with considerations	Comments
Ammonium sulfite caramel (E 150)	Brown colouring is a complex mixture of compounds obtained by heat treatment of carbohydrates (glucose, fructose, sucrose and/or invert sugar) with ammonia. The high-molecular-weight fraction can be composed of sugars, mainly pseudodisaccharides (anhydrous d-fructose dimers) and other monosaccharides depending on the origin source of carbohydrates (fructose, glucose or sucrose) [17, 18].
Arabic gum	Galactose polysaccharide is a mixture of polysaccharides and glycoproteins (galactans), composed of chains of D-galactose and D-glucuronic acid with terminal L-rhamnose and L-arabinose. It is a soluble fibre not digestible by intestinal enzymes or acidic environments [19].
Carrageenans	A mixture of copolymer esters of galactose and 3,6-anhydrogalactose with sulfates. Their absorption at the gastrointestinal level is minimal as it is not degraded by gastric pH or gut microbiota, although some absorption of the polysaccharide may take place and lower-molecular-weight fractions may be absorbed. They may include sugars for standardisation purposes [20].
Ethyl alcohol/ethanol	Alcohol should be used with caution due to possible liver damage and its potential endogenous fructose production [21].
Glucose, dextrose	High dose of glucose could be converted into fructose due to the polyol pathway [21].
Guar flour	A combination of galactose and mannose polysaccharide, it is a soluble fibre made up mainly of galactomannan, which consists of a linear chain of mannopyranose joined by glycosidic β linkage (1 ◊ 4) and branches of galactopyranose joined by α linkage (1 ◊ 6). It is not absorbed by the gastrointestinal tract [22].
Mannitol (E 421) (oral)	Fructose polyalcohol. Oral mannitol has an absorbance of 25–65%. In the liver, 7–10% of the absorbed mannitol can be transformed into fructose or oxidised to CO_2_ by unknown pathways [5, 23, 24].
Polydextrose	Polymer of glucose with glycosidic β linkage (1 ◊ 6) and sorbitol end groups (10%). In the human gastrointestinal tract, these links are resistant to hydrolysis by digestive acid and enzymes. However, the percentage of links that rupture is unknown [25].
Sucrose (intravenous)	Fructose and glucose disaccharide. Approximately 70–90% of the intravenous infusion dose is eliminated unaltered in urine as disaccharide, but there is substantial interindividual variability [26].
Starch syrup, glucose syrup	A mixture of glucose derivatives obtained from starch hydrolysis (glucose, maltose and oligosaccharides). Sometimes, part of the syrup is treated enzymatically, transforming some glucose into fructose to achieve greater sweetness [5]. Also, high dose of glucose could be converted into fructose due to the polyol pathway [21].
Tragacanth gum	A mixture of polysaccharides, mainly basorine and tragacanthin (water-soluble fraction of L-arabinose, L-fucose, D-xylose, D-galactose and D-galacturonic acid). Its degree of hydrolysis is unknown, giving rise to short-chain fatty acids [27].
Xanthan gum, corn sugar gum (E 415)	A combination of galactose and mannose polysaccharide, it is obtained by fermentation of carbohydrates (glucose or sucrose). It is mainly a salt of a high-molecular-weight polysaccharide containing D-glucose, D-mannose, D-glucuronic acid and pyruvic acid. It is not digestible by digestive enzymes and acidic medium [28].
Vanillin, vanilla flavouring	A flavour that contains fructose in its composition.

**Table 3:** Excipients not recommended for HFI patients

Not recommended excipients	Comments
Mannitol (E 421) (intravenous)	Fructose polyalcohol. Oral mannitol has an absorbance of 25–65%. In the liver, 7–10% of absorbed mannitol can be transformed into fructose or oxidised to CO_2_ by unknown pathways [5, 23, 24].

**Table 4:** Contraindicated excipients in HFI patients

Contraindicated excipients	Comments
Elderberry syrup	Flowers and elderberry fruits contain sugars, including mainly fructose and glucose with less sucrose [29].
Fructose	Monosaccharide. It is metabolised through aldolase B, the deficient enzyme in HFI.
Honey	Natural sweetener with 75% carbohydrates, of which fructose is the main sugar (38%). It also contains glucose (31%) and maltose or sucrose (< 5%)
Invert sugar	A mixture of glucose and fructose in equal parts.
Isomaltitol, isomalt	A mixture of polyalcohols: sorbitol ≤ 6%, mannitol ≤ 3%, maltitol and glucose-mannitol. The disaccharides are hydrolysed in the intestine in small quantities (approximately 10%), and the sorbitol released is only partially absorbed; the rest is degraded by the gut microbiota [23].
Maltitol, maltitol syrup, Lycasin, hydrogenated glucose syrup	Polyalcohol: glucose-sorbitol disaccharide. Glucose and sorbitol are hydrolysed in the intestine (approximately 40%), but the sorbitol released is only partially absorbed; the rest is degraded by gut microbiota [23].
Raspberry syrup	Concentrated raspberry syrup contains fructose.
Sorbitol, sorbitol syrup	Polyalcohol. The 25% absorbed in the intestinal tract is converted into fructose by sorbitol dehydrogenase.
Sorbitol esters	Esters are hydrolysed to fatty acids and sorbitol anhydrides in the gastrointestinal tract (the proportion will depend on the type of ester, vehicle, etc.) [30, 31].
Sucrose, sugar, saccharose, simple syrup (oral)	Fructose and glucose disaccharide. In the small intestine, the linkage is hydrolysed by glucosidase, and the released fructose is absorbed.
Sucrose esters	Esters are hydrolysed at a proportion of 70–80% in the human gastrointestinal tract, releasing sucrose [32].
Thyme syrup	Syrup with an unknown composition.

Some excipients classified as suitable in HFI were galactose and lactose, glucose starches (glucose polysaccharides), artificial sweeteners (aspartame, acesulfame, cyclamate and saccharin), nonsorbitol polyalcohols (xylitol), glycerol esters, propylene glycol esters, polysorbates (tweens), gelatines, colouring agents, waxes, vegetable oils, acids, alcohols, proteins, fatty acids, ions, and salts.

Glucose syrup was classified as a suitable excipient in HFI with some considerations because although it is a suitable sweetener, it may contain fructose and also due to its potential endogenous metabolism into fructose [21]. In the first revision, corn syrup was considered a contraindicated excipient because the term was used to refer to high-fructose corn syrup (HFCS), but this was corrected in later versions of the Spanish Medicines Agency database due to our intervention with this Agency [33]. Other mistakes were corrected, such as hydrogenated starch (maltitol, sorbitol synonymous) and glucose-fructose syrup, which were both classified in the first list as contraindicated when they were labelled “pregelatinised starch” and “glucose syrup” respectively.

Furthermore, when active ingredients were the same compound as excipients, the apps classified them with the same criteria. For example, in HFI, intravenous mannitol was classified as not recommended, and lactitol was classified as contraindicated, while glucose was classified as not recommended in diabetes mellitus. Furthermore, sucrose and isomaltitol irons were classified as suitable with considerations (suitable*) for HFI patients due to their unknown metabolism.

For the other related intolerances analysed, the classification was as follows: wheat starch was contraindicated and oatmeal was not recommended in celiac disease; oral lactose and lactose-based coprocessed excipient (Cellactose®) were contraindicated in lactose intolerance; and glucose, invert sugar and oral sucrose were classified as not recommended in diabetes mellitus, while some other monosaccharides were suitable with considerations (galactose, lactose).

### Application design and usability

**Fig. 1:**
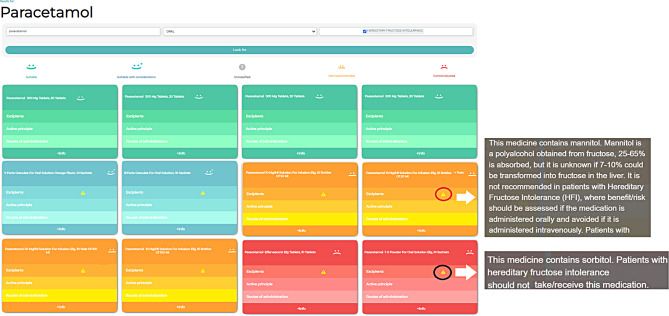
Example of results screen of IntoMed® applications

The mobile app was named IntoMed® (available for Android® and iOS®), and the website was located at https://checkmedicine.fiibhuilhuse.es/. These tools allow users to search for drugs through several methods: intolerance selection (one or more intolerances can be selected), searcher (search can be made by active ingredient, trademark name, national drug code or excipient) and route of administration (all routes, oral, parenteral or topical). The results are shown in order of tolerability (suitable drugs appear first and contraindicated drugs at the end), and a note is displayed detailing the classified excipients (except those classified as suitable) (Fig. 1). The mobile version allows intolerances and favourite drugs to be stored. In addition, an information section shows the national drug code, anatomical therapeutic chemical classification, need for medical prescription, and generic or nongeneric drugs, and the Spanish labelling and package leaflet can also be downloaded. A help section is included where the methodology used to classify medicines according to their excipients is explained. Legal, privacy, cookies and data protection policies (apps do not collect personal data) according to current legislation were also included.

The update of the apps with the Spanish Medicines Agency database shall be done at least twice a year. In the event of a major discrepancy being detected, it may be carried out at any time. Minor updates or development improvements will be carried out continuously through an annual maintenance contract with the informatics company.

The total usability result was 82.07 ± 13.46 points in 35 users (median age: 43 ± 10 years old); therefore, the tool is considered to have good usability. Among 12 health professionals, the result was 74.38 ± 13.94 points (median age: 42 ± 11.07 years old), and in patients and family members, it was 86.09 ± 11.55 points (median age: 44 ± 9.5 years old). Comments about usability were used to improve the experience before the apps were launched.

## Discussion

The IntoMed® tools designed in this study provide, in a fast and accessible way, reliable information about the tolerability of different drugs according to their excipient content in patients with HFI and other related intolerances. Despite the progress in drug labelling, questions related to drug and sweetener tolerance (which in many cases are also excipients) are still frequent in HFI patients and their families [34].

European medicine legislation contraindicates fructose, sucrose, invert sugar, sorbitol, maltitol, lactitol and isomaltitol because they can have serious or even fatal consequences if they are administered to HFI patients. In the last legislation update [6], it was established that a warning for oral or parenteral (not intravenous) products with fructose and sorbitol at dosages of less than 5 mg/kg/day did not need to be included. This means that an oral drug with less than 5 mg/kg/day of fructose or sorbitol will not have a warning, while a drug with the same amount of contraindicated polyalcohols will, which is inconsistent considering polyalcohol metabolism. Polyalcohols undergo incomplete hydrolysis by disaccharidases in the gastrointestinal tract; for example, partial hydrolysis occurs in approximately 40% of maltitol (and sorbitol is partially absorbed), approximately 10% of isomaltitol and approximately 2% of lactitol [5, 23]. Although these polyalcohols are not permitted in HFI patients, the total absorbed fructose (sorbitol) would be less than the amount of fructose that is currently allowed by the legislation. The Italian Health Institute has been more restrictive, and it has established a fructose or sorbitol threshold of 2.4 mg/kg/dose for parenteral products and vaccines in HFI patients [35]. For this reason, the apps include any difference in the classifications of parenteral non-IV and IV drugs, as well as to simplify searches for users. Mannitol is not considered a contraindicated polyalcohol in the legislation; however, it has been classified as not recommended in this review. This decision is derived from its unknown metabolism. Mannitol, a fructose polyalcohol, is metabolised in the liver at approximately 7–10% and can be transformed into fructose or into CO_2_ by an unknown metabolic pathway [23, 24]. In 10% or 20% intravenous mannitol solutions, the amount provided is very high, 50–100 g/500 ml, respectively, and the consequences of its administration are unknown, which is the reason for its not recommended classification.

On the other hand, intravenous sucrose is not contraindicated because after its administration, the blood glucose concentration does not increase, and it is mainly excreted in the urine as a disaccharide [26] (because there is no disaccharidase activity outside the gut). The absence of this enzymatic activity outside the gut explains why there is no warning for parenteral sucrose formulations in the current legislation, but there are doubts about its tolerability among some health care professionals [35–37].

Glucose has always been the recommended sweetener in HFI patients. However, a recent study in aldolase B knockout mice has demonstrated that high glucose consumption in these mice can activate the polyol pathway and produce fructose from glucose (glucose to sorbitol by aldolase reductase, and sorbitol to fructose by sorbitol dehydrogenase), resulting in liver alterations and growth delay [21]. Further studies in humans with HFI are needed to understand the implications and consequences of fructose consumption, establishing recommended limits and/or contraindications. For this reason, glucose and its derivatives have been designated as “suitable with considerations”, but these recommendations will be updated as more evidence becomes available.

Serious errors in some sweetener definitions have been found in technical data sheets, especially in glucose derivatives. Hydrogenated starches (a synonym for polyalcohols) are obtained by a partial transformation of glucose into sorbitol, and there has been confusion between starch (suitable) and hydrogenated starch (contraindicated). On the other hand, pregelatinised or modified starch has been confused with hydrogenated starch, but in modified starch, part of its glucose chains are broken to obtain variations in its viscosity without fructose or sorbitol content. Additionally, corn syrup should be a synonym of glucose syrup, but sometimes, to increase the sweetening power of corn syrup, part of the starch or glucose is isomerised to fructose by glucose isomerase, obtaining HFCS (glucose-fructose syrup). Some confusion might appear around the term corn syrup because it has been incorrectly used to refer to both glucose syrup and HFCS [33]. Differences between food and drug legislation in relation to glucose and fructose syrup have been found. Food legislation defines glucose syrup as glucose products with up to 5% fructose, calling them “glucose and fructose syrup” only when the fructose is greater than 5% [38]. However, drug legislation states that if the glucose syrup contains fructose, it should be indicated in the ingredient list [6], but this has not always been done.

Gums and colloidal polysaccharides were classified as suitable (with considerations) in the apps in HFI patients, although some controversy has been found concerning their composition. Gums and other colloidal polysaccharides (carrageenan type) are not hydrolysed at the gastrointestinal level, and their absorption will be minimal. However, some of them may include sugars to improve their quality, or they may have been obtained through glucose or sucrose fermentation. For this reason, it is necessary to be cautious until more information is provided [19, 20, 22, 27, 28].

In current medicine legislation, aromas and flavours can be declared in generic terms, although components with a recognised action or effect must be specifically declared [6]. Supplements are added to flavours to facilitate storage, standardisation, dilution, dissolution and/or stabilisation, and they may be sugars or polyalcohols that are contraindicated in HFI (sorbitol, isomaltitol, maltitol or lactitol) [39]. Furthermore, there are flavours that contain fructose, sucrose or sorbitol, and according to HFI dietary guidelines, they are not recommended (e.g., honey flavouring, vanillin, vanilla flavouring, liquorice extract, etc.) [4]. Even so, there are fruit juices or extracts with unknown compositions, colours obtained by mechanical methods from fruit (beet red) or sugar derivatives (caramel colour). In these cases, there is no warning for HFI patients on the labels.

Sucrose esters or sorbitol esters (spans) obtained by the partial esterification of sorbitol with fatty acids are hydrolysed by esterases and release sucrose or sorbitol [30–32]. Spans are usually present in topical preparations and in this case are considered suitable [40, 41], but when they are used in oral or parenteral drugs, caution is needed in HFI patients. In contrast, polysorbates (tweens) are sorbitol esters with polyoxyethylene fatty acids, but the link between sorbitol and ethylene oxide cannot be hydrolysed [30]. Despite this, polysorbate intoxication after the administration of an intravenous drug with polysorbate 80 was described in an HFI patient with hepatorenal alterations and hypoglycaemia, similar to fructose intoxication [42]. There are also studies describing liver and kidney toxicity in non-HFI patients taking drugs containing polysorbates [43], so it is possible that the alterations were caused by the toxicity of polysorbate itself rather than fructose intoxication.

There is increasing interest in electronic health (eHealth), especially in mobile technology (mHealth), in recent years, with the aim of helping patients, consumers and even health professionals. These technologies could be useful due to the lack of information on rare diseases [7, 44] and because many health professionals encounter few patients with these characteristics throughout their professional career, and therefore, they require the help of decision-making tools. These apps contain information not only about excipients with box warnings but also about some other sugars, sweeteners or excipients for which there is currently no specified legislation for HFI patients but that may produce intolerance when they are present in drugs. In addition, the possibility of selecting other diseases was included in the development of the apps, as HFI has also been linked with other pathologies, such as type 2 diabetes mellitus or glucose intolerance [8, 9] and celiac disease [10]. The usability of digital applications by potential users is a key parameter of good practice in their development. In our case, the reliability and usability of the tools were demonstrated, although the score was better in patients than in health professionals, possibly due to the problems they encountered daily. Furthermore, the sample used for this study consisted of a multidisciplinary team (included in the design), HFI patients and their families, and health professionals. The SUS is a frequently used questionnaire in eHealth and mHealth because it is an easy and quick tool [45] and is validated in Spanish [15], although it was not designed to evaluate the usability of mHealth apps.

The present study has several limitations. The information about excipient metabolism described in the literature is old and incomplete, and it is not specific for HFI patients. For this reason, in some cases, it is not possible to obtain conclusions about metabolism or composition related to fructose. Moreover, the information for classifying excipients in these apps depends on updates from the Spanish Medicine Agency, so a warning is included for users.

In conclusion, IntoMed® is a new tool for finding information about the tolerability of drugs containing excipients in patients with HFI and other related intolerances. It is a fast and reliable system that covers the current excipient legislation and expands it with other specific information. It is important to highlight that there are excipients not included in the legislation and do not appear on labels; however, due to their metabolic involvement, they should have a warning for HFI patients. These excipients include mannitol (especially in IV drugs), fruit syrups, honey, sulfite caramel and vanilla. Moreover, it is necessary to take special precautions with glucose and glucose syrups because of frequent and important errors in compounding and their potential endogenous production of fructose. Through the review carried out in this study, the deficiencies of some excipients with unclear information have been revealed. Furthermore, it has been possible to respond to these unmet needs.

## Data Availability

The datasets generated and/or analyzed during the current study are not publicly available due individual privacy but are available from the corresponding author (Izquierdo-García E: elsa.izquierdo@salud.madrid.org) on reasonable request.

## Abbreviations

HFI: Hereditary Fructose Intolerance
app: Application
IV: Intravenous
HFCS: High fructose corn syrup
NAFLD: Non-alcoholic fatty liver disease
eHealth: Electronic Health
mHealth: Mobile Health
SUS: System Usability Scale
